# Cross-Modal Integration of Reward Value during Oculomotor Planning

**DOI:** 10.1523/ENEURO.0381-19.2020

**Published:** 2020-02-07

**Authors:** Felicia Pei-Hsin Cheng, Adem Saglam, Selina André, Arezoo Pooresmaeili

**Affiliations:** Perception and Cognition Group, European Neuroscience Institute Goettingen-A Joint Initiative of the University Medical Center Goettingen and the Max-Planck-Society, Goettingen 37077, Germany

**Keywords:** reward, saccade, cross-modal integration, audiovisual

## Abstract

Reward value guides goal-directed behavior and modulates early sensory processing. Rewarding stimuli are often multisensory, but it is not known how reward value is combined across sensory modalities. Here we show that the integration of reward value critically depends on whether the distinct sensory inputs are perceived to emanate from the same multisensory object. We systematically manipulated the congruency in monetary reward values and the relative spatial positions of co-occurring auditory and visual stimuli that served as bimodal distractors during an oculomotor task performed by healthy human participants (male and female). The amount of interference induced by the distractors was used as an indicator of their perceptual salience. Our results across two experiments show that when reward value is linked to each modality separately, the value congruence between vision and audition determines the combined salience of the bimodal distractors. However, the reward value of vision wins over the value of audition if the two modalities are perceived to convey conflicting information regarding the spatial position of the bimodal distractors. These results show that in a task that highly relies on the processing of visual spatial information, the reward values from multiple sensory modalities are integrated with each other, each with their respective weights. This weighting depends on the strength of prior beliefs regarding a common source for incoming unisensory signals based on their congruency in reward value and perceived spatial alignment.

## Significance Statement

Real-world objects are typically multisensory, but it is not known how reward value is combined across sensory modalities. We examined how the eye movements toward a visual target are modulated by the reward value of audiovisual distractors. Our results show that in the face of uncertainty as to whether co-occurring visual and auditory inputs belong to the same object, congruence in their reward values is used to guide audiovisual integration. However, when a strong prior exists to assume that unisensory inputs do not emanate from the same object, the associative value of vision dominates over audition. These results demonstrate that our brain uses a reward-sensitive, flexible weighting mechanism to decide whether incoming sensory signals should be combined or not.

## Introduction

Sensory perception is not merely driven by the incoming sensory inputs but is also affected by top–down information (Gilbert and Sigman, 2007). Among the top–down influences on perception, the effect of reward is particularly important to motivate an agent, facilitate learning, and help the agent to behave adaptively given the limited capacity of both sensory and motor systems. It has been shown that reward acts to modulate selective attention when the subject’s performance was directly linked to the monetary reward (Small et al., 2005; Mohanty et al., 2008; Engelmann et al., 2009), even when the monetary reward was no longer task relevant (Della Libera and Chelazzi, 2006; Hickey and van Zoest, 2012; Pooresmaeili et al., 2014; Asutay and Västfjäll, 2016; Luque et al., 2017). Rewards may act as guiding signals for learning and optimizing specific attentional operations (Chelazzi et al., 2013), and not only can increase the salience of associated stimuli (Hickey and van Zoest, 2012), but also enhance the suppression of the distractors (Della Libera and Chelazzi, 2009), change the priority maps of space (Chelazzi et al., 2014), and reduce the intrinsic neural noise in the motor and cognitive control (Manohar et al., 2015).

Nevertheless, although there is a plethora of studies on various aspects of reward, to date evidence showing the effect of reward association in one sensory modality on the perception of another modality and, more importantly, how reward from different sensory modalities interact with each other remains scarce (but see Leo and Noppeney, 2014; Pooresmaeili et al., 2014). This is despite the fact that in natural environments objects are typically multisensory and comprise multiple attributes that could potentially have either similar or distinct associative values, which underscores the importance of understating how information related to reward value is integrated across senses. From a sensory integration point of view, it is well known that our brain combines multiple sensory signals based on cue integration principles into coherent percepts to reduce uncertainty (Ernst and Banks, 2002; Kersten et al., 2004; Knill and Pouget, 2004) or use information in one sensory modality to prioritize information processing in another sensory modality that better serves as an “expert system” according to the task at hand (Macaluso and Driver, 2005). In other cases, however, perception may be dominated by information from one sensory modality, with other modalities being partially or completely disregarded (Colavita, 1974; Spence, 2009).

Critically, in addition to the physical characteristics of the input stimuli, such as their spatial or temporal properties, recent studies also provided evidence for the effect of cognitive factors on audiovisual integration. For example, expectations of stimulus characteristics can reduce reaction times in a task that requires audiovisual integration (Van Wanrooij et al., 2010; Zuanazzi and Noppeney, 2018), and emotional (Maiworm et al., 2012) and motivational (Bruns et al., 2014) factors can both influence cross-modal binding processes.

Together, the physical and cognitive characteristics of multisensory objects have been shown to affect the processing of information across senses. Although several computational models have been put forward to explain the principles governing cross-modal processing based on the physical characteristics of stimuli (Stein and Meredith, 1993; Calvert, 2001; Stein and Stanford, 2008; Chandrasekaran, 2017), the role of cognitive factors and their possible interactions with physical stimulus characteristics has remained underexplored.

In the present study, our aim was to examine the effect of (1) associated reward values and (2) spatial alignment of auditory and visual signals in the integration of reward value across sensory modalities. We systematically manipulated these factors during the course of the following two experiments: experiment 1 manipulated the congruence of reward values between the auditory and visual stimuli, and experiment 2 manipulated both reward value congruency and spatial congruency.

The experiments were based on a visually driven saccade task that involved oculomotor interference created by a distractor. Saccades provide a reliable readout of cross-modal interactions, and previous studies have investigated the influence of bimodal targets or distractors on saccade planning (Corneil et al., 2002; Doyle and Walker, 2002; Campbell et al., 2010; Heeman et al., 2016). Importantly, the transiently associated reward value of a visual distractor has been shown to modulate the magnitude of its interference with the planning of the saccades to a target (Hickey and van Zoest, 2012). We hypothesized that in such a task that highly relies on the processing of visual spatial information, visual rewards dominate the effect of bimodal distractors on saccade planning but reward value is integrated across senses if there is ambiguity as to whether unisensory auditory and visual inputs have a common source or not.

## Materials and Methods of experiment 1

### Participants

Twenty-four participants (18–34 years old; mean age = 25.0 years, SD = 4.0; 11 males) took part in study 1. One subject was excluded because >25% of trials had to be discarded (see the following analyses paragraph for the trial exclusion criteria). All participants were without any neurologic or psychiatric disorders and had no recent use of drugs, medications, or alcohol dependence, and all had normal or corrected-to-normal sight and normal hearing. Participants gave written informed consent after the experimental procedures were clearly explained to them. They received basic payment plus the payment that was proportional to the accumulated reward value they gained during the reward association part of the experiment. The experiment took ∼1.5 h, and participants were compensated by €14. The study was conducted in full accordance with the Declaration of Helsinki and was approved by the local Ethics Committee of the Medical University Goettingen (proposal 15/7/15).

### Apparatus

Eye movements were measured using the EyeLink 1000 eye tracker system (SR Research) in a desktop mount configuration, recording the right eye, with a sampling rate of 1000 Hz. The visual stimuli were presented on a calibrated LCD monitor with a refresh rate of 120 Hz, and the auditory stimuli were presented with over-ear headphones (HAD 280 audiometry headphones, Sennheiser). Stimuli were produced by MATLAB and the Psychtoolbox with custom-made scripts.

### Materials and procedure

**Figure 1.**
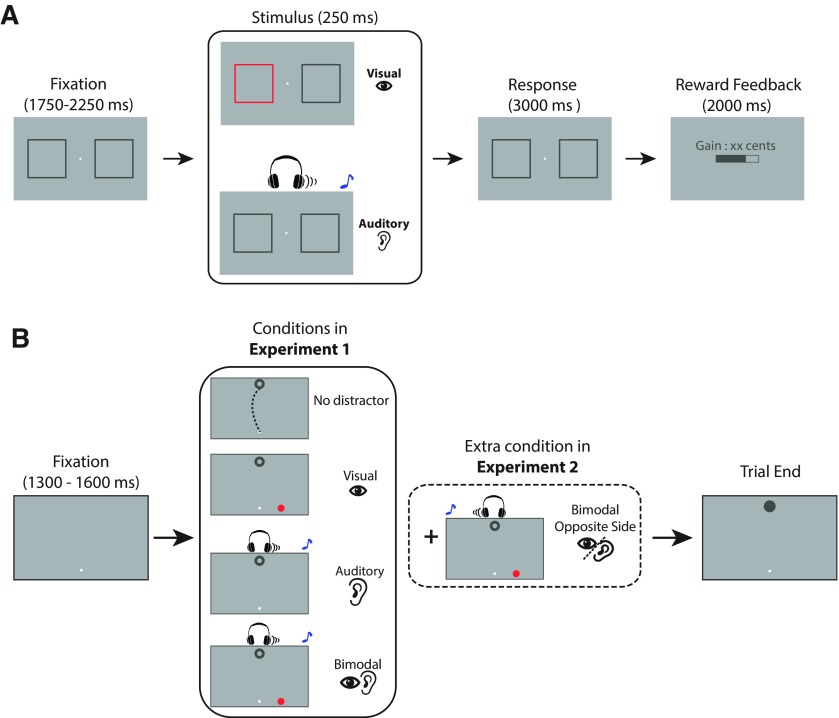
Behavioral tasks. ***A***, Reward-conditioning task: participants learned the reward associations of two colors and to sounds through a pavlovian conditioning paradigm. They were instructed to indicate the side of a sound or a color change by key presses. Correct answers received a nonzero reward, the amount of which depended on the preceding sound pitch (600 or 1000 Hz) or color (magenta or mustard). The reward was displayed graphically as the filled area of a bar as well as a number that corresponded to the amount of monetary reward in Euro cents. Incorrect answers received a reward of zero. ***B***, Saccadic task in the postconditioning part of experiments 1 and 2: participants were instructed to make an eye movement from the fixation point to the target (a ring). In each trial, the participant started by maintaining eye position on the fixation point for a random duration between 1300 and 1600 ms before a target circle was presented either above or below the fixation point, simultaneously with one of the following conditions: (1) with no distractor; (2) with a visual distractor either to the right or left side of the horizontal position of the fixation point; (3) with an auditory distractor presented on the right or left side of the headphone; and (4) with a bimodal distractor, which was composed of both the visual and auditory distractors presented on the same side relative to the fixation point. In experiment 2, one more condition was included, in which the auditory distractor was presented on the opposite side relative to the visual distractor.

Participants were comfortably seated in a dimly lit room, with their head rested on a chinrest. The experiment included a pavlovian conditioning part to familiarize the participants with the reward associations, and a postconditioning part that required the participant to perform saccadic eye movements after they had learned the reward associations (Fig. 1). Each participant performed one block consisting of 120 trials in the conditioning part, 9 training trials to familiarize with the task needed for the postconditioning part, and five blocks consisting of 135 trials (15 repetitions of each of the nine experimental conditions) for the postconditioning part. Participants were allowed to take a break for exactly 3 min between the blocks. The eye tracker was calibrated at the beginning of the experiment and after each break by the participant fixating at 13 randomly presented targets spanning the display (EyeLink calibration type HV13).

In the conditioning part, participants learned the reward associations of two sounds (600 or 1000 Hz, sawtooth waveform) and two colors (light magenta and light mustard colors, RGB values [171, 136, 0] and [239, 77, 255]) by performing a localization task (Fig. 1*A*). The loudness level of the sounds and the luminance of the two colors had also been equalized (55 dB and 70 cd/m^2^, respectively), and the background color was mid-gray (RGB: [128, 128, 128]).

At the beginning of each trial, the participant was instructed to maintain eye position on a dot (0.3°) at the center of the screen for 1750–2250 ms. The stimulus display also contained two square-shaped frames to the left and right side of the fixation point (12° eccentricity, 10° size, RGB: [80, 80, 80] with a luminance of 70 cd/m^2^). After the fixation period, either a sound (600 or 1000 Hz, counterbalanced across trials) was played on the right or left side of a headphone or a color change occurred in one of the two square frames. All sounds and colors were presented for a fixed duration of 250 ms. Participants were instructed to indicate the correct side of the sound or the color change by pressing the right or left arrow on the keyboard within 3.25 s from the onset of the sounds or colors. Participants received feedback about the amount of obtained reward in each trial, presented graphically as the filled area within a bar as well as a sentence indicating the amount of reward in Euro cents (all with a dark gray color, RGB: [80, 80, 80]). Incorrect answers led to a reward of zero, whereas correct answers were rewarded by a number drawn from a normal distribution, with high/low mean reward values of 24/4, and an SD of 0.5. The pairing of reward (high or low) with the sound or color was counterbalanced across subjects. Presentation of each modality (sound/color), reward (high/low), and side (right/left side relative to the fixation point) was pseudorandomized for each subject, and was programmed in a way that none of them repeated consecutively for more than three trials. At the end of the conditioning part, the participant was required to indicate the sound and the color that gave higher reward by pressing number “1” or “2” on a keyboard. The orders for the presentation of the two sounds and two colors were both randomized. The auditory pure tones in both of our experiments had an intensity of 55 dB and frequencies of 600 or 1000 Hz. We decided to use these frequencies since they produce approximately the same loudness based on their location on equal-loudness contours (Robinson and Dadson, 1956). In our pilot experiments, we tested a range of sound frequencies but did not observe a difference in terms of the strength of saccade trajectory deviations induced by auditory distractors in the main saccade task. The intensity of the sounds was selected based our subjective estimation of a sound level that is most comfortable for the participants, but we did not explore the effect of different sound intensities in terms of their effect on saccade curvatures.

In the postconditioning part, we used a modified version of the task used by a previous study (Hickey and van Zoest, 2012), where participants had to make an eye movement from the fixation point to a target (Fig. 1*B*). The participant first pressed the space bar to begin a trial. Following 1000 ms of successful fixation (eye position maintained within 1° from the center of fixation point with a size of 0.3°), the fixation point shrank in size (0.15°) and a second fixation period that jittered between 300 and 600 ms had to be fulfilled before the presentation of the experimental stimuli. If eye position offsets >1° from the center of the fixation point were detected any time within the 1300–1600 ms of the designated fixation period, this interval started over from the beginning. Successful fixations were followed by the presentation of a target (a dark gray ring, RGB: [80, 80, 80], diameter = 1°) that was presented either 7.38° above or below the fixation point, simultaneously with one of the following conditions: (1) with no distractor; (2) with a visual distractor (colored circle with a radius = 0.8°) either 3.6° to the right or left side of the horizontal position of the fixation point; (3) with an auditory distractor presented on the right or left side of the headphone; and (4) with a bimodal distractor, which was composed of both the visual and auditory distractors presented. The visual, auditory, and bimodal distractors were based on the stimuli used in the conditioning part (i.e., the colors and sounds that had been associated with reward values). Thus, distractors comprised nine conditions: no distractor, visual (high or low reward), auditory (high or low reward), and bimodal (high or low visual × high or low auditory, four conditions in total). Each condition was repeated 75 times across all trials in a pseudorandomized sequence. Upon a saccade to the target (within 3° from the center of the target ring), the ring was filled in and stayed in view for another 100 ms before the disappearance of the target and distractors and the start of the next trial.

Same as the conditioning part, after each postconditioning block, the participant was also required to indicate the sound and the color that gave high reward by pressing the number 1 or 2 on the keyboard, and the orders for the presentation of the two sounds and two colors were both randomized. After the whole experiment, the participants had to fill out a questionnaire regarding the reward values of the stimuli and whether they had any preference toward any stimulus. This questionnaire was used to exclude participants that potentially had misunderstood reward associations (e.g., indicated that high reward sound or color was always presented on the right side).

### Data analyses

Data were processed and analyzed by using MATLAB (version R2015a). Trials from the training sessions were excluded from the analyses. For each trial, the first saccade after the target onset was analyzed. To detect the saccades, eye position samples in a trial were smoothed using a Savitzky-Golay lowpass filter with an order of 2 and length of 10 ms (Nyström and Holmqvist, 2010). Saccade onsets were defined as the moment when a sample exceeded an angular velocity threshold of 35°/s and an acceleration of 9500°/s^2^. Saccade offsets were calculated as the first sample where the eye position velocity and acceleration fell below the aforementioned thresholds.

Trials with a saccadic latency <80 ms or >400 ms, with a saccadic duration of >120 ms were excluded from the analyses. Additionally, trials where the angular deviation of the saccade end point or the saccade amplitude was >2.5 SDs away from the subjects’ grand mean (across all trials of the session) were defined as outliers and were removed from the analysis. 1 subject was excluded because >25% of trials were discarded based on these criteria. In the remaining 23 subjects, 94.78 ± 2.33% of trials were taken into the analysis.

**Figure 2.**
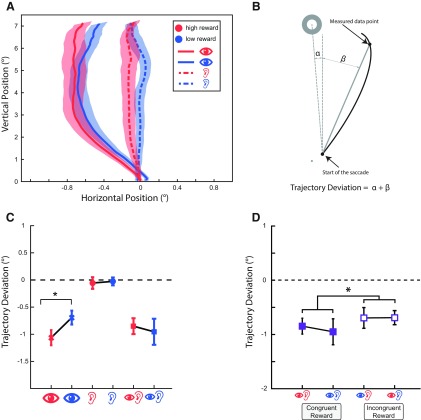
The effect of reward value on saccade trajectories in experiment 1. ***A***, Saccadic trajectories of the high- and low-reward visual and high- and low-reward auditory conditions (indicated by corresponding symbols and colors). ***B***, Calculation of the angular trajectory deviation of the saccades. ***C***, Baseline corrected, average angular deviations of saccade trajectories as shown in ***A*** and ***B*** served as a measure of oculomotor interference created by the visual, auditory, and bimodal distractors with either high or low reward value (only bimodal distractors with congruent reward values are shown in ***C***). Positive values indicate that the saccadic trajectories deviate toward the distractor, while negative values indicate deviations away from the distractor. Visual distractors associated with the high reward value resulted in significantly larger deviations away from the distractor, compared with low-value distractors. ***D***, Baseline corrected saccadic trajectory deviations in all bimodal conditions. From left to right: congruent-reward/both high-value, congruent-reward/both low-value, incongruent-reward/visual low-value and auditory high-value, and incongruent-reward/visual high-value and auditory low-value conditions. Saccade trajectories had significantly larger deviations when reward values were congruent compared with when they were incongruent across modalities. Error bars indicate the SEM. Asterisk corresponds to a significance level of *p* < 0.05.

Our statistical inferences and conclusions throughout are based on the mean angular deviation of saccade trajectories, i.e., similar to previous studies of the effect of reward on oculomotor salience (Hickey and van Zoest, 2012). The calculation of the angular deviation of the saccade trajectories for a typical trial is demonstrated in Figure 2, *A* and *B*. Angular deviation was defined as the angular distance between the line joining each sampled eye position along the saccade trajectory to the saccade starting point and the straight path between the saccade starting point and the target. The mean angular deviation is thus the average angular deviation of all samples along the saccade trajectory.

Since the target position could be either in the upper or lower hemifield, and the distractor could be either on the left or right side relative to the fixation point, to examine the angular deviation, we rectified the target position to the upper hemifield, and the distractor to the right side relative to the fixation point. In each participant, the no-distractor condition served as baseline, and their averaged angular deviation was subtracted from the average angular deviation of each distractor condition with the corresponding target location (i.e., in upper or lower hemifield). Even in the absence of distractors, saccades may exhibit trajectory deviations, which are predominantly toward one of the quadrants. Subtraction of the no-distractor trajectories remedies this directional bias. However, our estimation of the baseline using the no-distractor condition could be noisy. Therefore, the baseline was determined by averaging the angular deviation of no-distractor trials that were within a 68% confidence interval around the mean in this condition (absolute *z*-score, <1). We obtained the same statistical results as reported here, when the data were analyzed without the subtraction of the baseline.

We analyzed the saccade average angular deviations by carrying out a series of two-tailed, paired *t* tests and repeated-measures ANOVAs (RANOVAs). The first preplanned analysis examined whether the effect of reward on visual distractors observed in previous studies can be reproduced in our setup and in the case of stimuli tested during the nonreward phase. In a second analysis, we focused on the effect of reward across modalities (in cases where the reward was unambiguous; i.e., congruent reward for the bimodal conditions), with factors for modalities (visual, auditory, bimodal) and reward (high vs low). A third analysis focused on reward congruency in the bimodal distractors, with factors for visual reward (high vs low), and auditory reward (high vs low). For each RANOVA, a Mauchly sphericity test (default test in MATLAB) assessed whether the assumption of sphericity is violated. In case of a violation of sphericity, a conservative lower-bound correction was applied to the df and *p* values of the RANOVAs. Significant effects from the omnibus RANOVAs were further investigated by performing *post hoc* tests (using the *multcompare* function in MATLAB with a Bonferroni correction for multiple comparisons). Effect sizes in RANOVAs are reported as partial η^2^ ($\eta_{p}^{2}$) and in pairwise comparisons as Cohen’s *d* (Lakens, 2013).

Latencies of the saccades (the time between the onset of the target and distractors and the onset of the first saccade), saccade duration (the interval between the onset and the offset of a saccade), and the distance of the end point of the saccades from the target are also reported in Table 1-Table 4.

**Table 1:** Latencies, durations and the distances of the end points of saccades from the target in experiment 1

	Visual	Auditory	Bimodal/reward congruent	Bimodal/reward incongruent (high reward visual/low reward auditory)	Bimodal/reward incongruent (low reward visual/high reward auditory)	No distractor
Latency (ms) high reward	218.19 ± 23.39	188.33 ± 20.98	196.94 ± 20.72	197.07 ± 19.26	198.37 ± 19.58	211.27 ± 23.95
Latency (ms) low reward	217.41 ± 20.42	186.72 ± 20.42	201.26 ± 21.82
Duration (ms) high reward	44.38 ± 33.17	44.74 ± 3.37	43.91± 3.32	43.97 ± 3.24	44.63 ± 3.66	44.90 ± 3.51
Duration (ms) low reward	44.50 ± 3.32	44.16 ± 3.33	44.26 ± 3.76
Distance of end point from target (°) high reward	0.87 ± 0.27	0.89 ± 0.32	0.91 ± 0.29	0.9 ± 0.29	0.91 ± 0.36	0.86 ± 0.24
Distance of end pointfrom target (°) low reward	0.89 ± 0.28	0.91 ± 0.29	0.90 ± 0.23

**Table 2:** Latencies of saccades in experiment 2, before and after learning of reward associations

	Visual	Auditory	Same side/congruent reward/ bimodal	Same side/ bimodal (high reward visual/low reward auditory)	Same side bimodal (low reward visual/high reward auditory)	Opposite side/congruent reward/ bimodal	Opposite side bimodal (high reward visual/low reward auditory)	Same side bimodal (low reward visual/high reward auditory)	No distractor
Latency (ms) high reward Pre	202.08 ± 23.85	174.21 ± 19.01	182.02 ± 20.24	180.63 ± 20.33	182.20 ± 20.51	178.01 ± 19.93	180.60 ± 20.44	184.58 ± 21.80	193.30 ± 24.16
Latency (ms) low reward Pre	204.28 ± 25.95	173.35 ± 18.92	183.24 ± 20.83			184.19 ± 21.23
Latency (ms) high reward Post	180.64 ± 24.28	158.97 ± 18.10	164.01 ± 19.38	164.92 ± 17.52	166.62 ± 19.49	164.20 ± 18.73	164.68 ± 18.38	167.98 ± 19.78	193.30 ± 24.16
Latency (ms) low reward Post	182.53 ± 24.16	159.22 ± 18.2976	168.00 ± 20.63			166.95 ± 19.38

**Table 3:** Durations of saccades in experiment 2, before and after learning of reward associations

	Visual	Auditory	Same side/congruent reward/ bimodal	Same side/bimodal (high reward visual/low reward auditory)	Same side bimodal (low reward visual/high reward auditory)	Opposite side/congruent reward/ bimodal	Opposite side bimodal (high reward visual/low reward auditory)	Same side bimodal (low reward visual/high reward auditory)	No distractor
Duration (ms) high reward Pre	43.93 ± 4.00	43.73 ± 4.18	43.65 ± 4.25	43.91 ± 3.99	43.68 ± 4.29	43.75 ± 4.03	43.57 ± 3.93	43.88 ± 4.09	44.05 ± 4.14
Duration (ms) low reward Pre	43.88 ± 4.17	43.74 ± 4.30	43.60 ± 4.09			43.92 ± 4.11
Duration (ms) high reward Post	43.51 ± 3.31	43.08 ± 3.76	43.32 ± 3.62	43.15 ± 3.38	43.35 ± 3.70	43.40 ± 3.94	43.34 ± 3.44	43.35 ± 3.77	43.78 ± 3.75
Duration (ms) low reward Post	43.66 ± 3.96	43.53 ± 4.16	43.62 ± 4.06			43.60 ± 3.84

**Table 4:** The distances of saccade end points from the target in experiment 2, before and after learning of reward associations

	Visual	Auditory	Same side/congruent reward/ bimodal	Same side/ bimodal (high reward visual/low reward auditory)	Same side bimodal (low reward visual/high reward auditory)	Opposite side/congruent reward/ bimodal	Opposite side bimodal (high reward visual/low reward auditory)	Same side bimodal (low reward visual/high reward auditory)	No distractor
Distance of end point from target (°) high reward Pre	0.86 ± 0.19	0.85 ± 0.15	0.87 ± 0.17	0.88 ± 0.19	0.84 ± 0.18	0.88 ± 0.19	0.85 ± 0.16	0.89 ± 0.16	0.83 ± 0.17
Distance of end point from target (°) low reward Pre	0.85 ± 0.16	0.85 ± 0.17	0.86 ± 0.17			0.88 ± 0.19
Distance of end point from target (°) high reward Post	0.91 ± 0.22	0.88 ± 0.2	0.91 ± 0.22	0.88 ± 0.2	0.92 ± 0.22	0.92 ± 0.21	0.89 ± 0.19	0.91 ± 0.22	0.89 ± 0.20
Distance of end point from target (°) low reward Post	0.91 ± 0.23	0.90 ± .20	0.91 ± .21			0.92 ± .20

## Results of experiment 1

### Conditioning task

In the conditioning part, participants only had to identify the location of the presented color change/sound in each trial. The mean correct responses were 99.6% across 120 trials, with an SD of 0.6% indicating that subjects could accurately localize the colors/sounds. All subjects had learned the reward value associated with each color and sound (based on their responses at the end of conditioning block and the questionnaire data).

### Saccadic task

#### The effect of reward value of visual distractors on saccade trajectories

We first examined whether we obtained results similar to those of previous studies (Hickey and van Zoest, 2012) for visual distractors. A preplanned, paired-samples *t* test on high versus low visual reward showed a significant effect (*t*_(22)_ = 2.63, *p* = 0.0152, Cohen’s *d* = 0.54), with high visual reward condition showing greater deviations compared with low reward (mean ± SD: −1.063 ± 0.68 and −0.693 ± 0.62 for high and low visual reward, respectively; Fig. 2*A*,*C*), similar to the results observed previously (Hickey and van Zoest, 2012).

#### Reward association across different modalities

For the analysis of the effect of reward across all modalities, we only took the conditions where the distractors carried unambiguous reward values (i.e., only reward congruent conditions were included; Fig. 2*C*). A two-way RANOVA with modality and reward as independent factors revealed a main effect of modality (*F*_(2,44)_ = 28.57, *p* = 0.23 × 10^−5^, lower bound corrected, $\eta_{p}^{2}$ = 0.56). *Post hoc* tests revealed significant differences between trajectory deviations of visual and auditory (mean ± SD: −0.88 ± 0.56 and −0.04 ± 0.36 for visual and auditory, respectively, *p* = 1.1 × 10^−5^, Cohen’s *d* = 1.27) and auditory and bimodal (mean ± SD: −0.04 ± 0.36 and −0.9 ± 0.79 for auditory and bimodal, respectively, *p* = 1.1 × 10^−5^, Cohen’s *d* = 1.16) conditions, but the difference between visual and bimodal conditions was not significant (*p* > 0.1, Cohen’s *d* = 0.05). The effect of reward (*F*_(1,22)_ = 0.75, *p* = 0.39) and the interaction between reward and modalities (*F*_(2,44)_ = 2.53, *p* = 0.12) did not reach statistical significance.

#### Reward congruency

For the analysis of reward congruency, we took all bimodal conditions and performed a RANOVA with visual reward (high or low) and auditory reward (high or low) as independent factors. Note that in this analysis our bimodal conditions are represented as high/high, low/low, low/high, and high/low, with respect to visual or auditory rewards, with congruent conditions represented as high/high or low/low (Figs. 1*B*, 2*D*). The results of RANOVA (lower bound corrected) showed a significant interaction between the visual and auditory rewards (*F*_(1,22)_ = 4.72, *p* = 0.040, $\eta_{p}^{2}$ = 0.17), indicating a significant effect of reward congruency. As can be seen in Figure 2*D*, saccadic trajectories exhibited stronger deviations away from the distractor when the visual and auditory reward values were congruent (mean ± SD = −0.90 ± 0.79) compared with when they were incongruent (mean ± SD = −0.69 ± 0.66). None of the main effects reached statistical significance (all *F* values < 1 and all *p* values > 0.5).

Having shown that learned reward associations of distractors affect the trajectory of visually guided saccades, in the following experiment 2 we further examined the interaction between cognitive factors (reward values of the visual and auditory components of the bimodal distractors) and the physical factors (spatial alignment of the visual and auditory components) of the distractor in a similar setup. Furthermore, we included a preconditioning part of the experiment to confirm that the effect of reward exists only after associative learning.

## Materials and Methods of experiment 2

### Participants

The number of the participants was calculated based on the results of experiment 1 (a two-tailed, paired *t* test comparing visual high versus low reward value conditions, α = 0.05, β = 0.8; the total number of participants required for this power was 32). In total, 36 participants were recruited, 4 participants were excluded (2 participants did not give correct answers to the questions regarding reward association right after the conditioning part, and were replaced by 2 new participants; 1 participant did not complete the second session; and 1 participant had poor calibration values). The final sample comprised 32 participants (age range, 19–37 years; mean age = 25.3 years, SD = 4.0; 11 males). The experiment was conducted on 2 consecutive days. On the first day, all participants received €17; on the second day, all participants received basic payment plus the payment that was proportional to the accumulated value they gained during the reward association part of the experiment (with a maximum of €31). All procedures related to the recruitment of the participants were identical to those in experiment 1 and complied with the ethical guidelines as described for that experiment.

### Apparatus

Identical to those used in experiment 1.

### Materials and procedure

The conditioning tasks used for the learning of reward associations and the saccadic task of experiment 2 were identical to those of experiment 1 (Fig. 1), but the procedures and experimental conditions were modified as follows. Experiment 2 was conducted on 2 consecutive days. On the first day of the experiment, participants first performed a saccadic localization task in which they indicated the location of the stimuli (visual; auditory; or bimodal, with sounds on the same or opposite sides relative to the visual stimulus) by making an eye movement toward their perceived locations. This calibration task allowed us to estimate the perceived location of the target and distractors that were used in the subsequent main saccadic task. Visual stimuli were presented 7.38° above or below the fixation point (same as the target location in the saccade task) or 3.6° to the right or left side of the fixation point (same as the distractor location in the saccade task), whereas auditory and bimodal stimuli were presented either to the left or to the right side of the fixation point (same as the distractor locations in the main saccadic task). In total, data on 10 training trials and 200 experimental trials, comprising 20 trials per condition, and location were collected. The stimuli were identical to those used in the later saccade task (and the same as those used in experiment 1), with the exceptions that the color of the disks was always dark gray (RGB: [80, 80, 80], the same as the target color in experiment 1, and the sound had a pitch (800 Hz) that was different from those used in the later parts.

The saccadic localization task was followed by a preconditioning part that required the participant to perform the saccadic task before the learning of the reward values (Fig. 1*B*). Each participant performed 13 training trials to familiarize with the saccade task followed by 10 blocks consisting of 150 trials for the preconditioning part (i.e., 1500 trials in total). In the saccadic task, the distractor conditions were one of the following: (1) no distractor; (2) with a visual distractor (0.8°) either 3.6° to the right or left side of the horizontal position of the fixation point; (3) with an auditory distractor presented on the right or left side of the headphone; (4) with a bimodal distractor, which is composed of both the visual and auditory distractors presented on the same side; and (5) with a bimodal distractor, which is composed of both the visual and auditory distractors presented on the opposite side (Fig. 1*B*). Note that visual and auditory distractors could have been associated with either high or low reward values. Hence, bimodal distractors comprised four conditions with respect to the associated reward value in each modality (i.e., both modalities high, both modalities low, vision high and auditory low, and vision low and auditory high reward values). Since bimodal distractors could be on the same or opposite sides, this resulted in a total of eight bimodal conditions (i.e., four reward pairings × two sides). Therefore, experimental stimuli comprised 13 conditions, each repeated 120 times (except for the no-distractor condition, which was repeated for 60 trials). The number of trials per distractor condition was increased compared with experiment 1 (120 trials per distractor condition in experiment 2 compared with 75 trials per condition used in experiment 1) to have a more robust estimation of the deviations in the trajectory of the saccades.

On the second day of the experiment, participants first performed the reward conditioning task (160 trials; i.e., 40 trials per reward condition in each modality), followed by the saccadic task (postconditioning part, same procedure, and number of trials as the preconditioning part). At the end of the session on day 2, participants performed the saccadic localization task where they indicated the perceived location of the experimental stimuli with eye movements again (10 training trials and 200 trials of the main task identical to day 1). This was to test whether training on the saccade task alters the perceived location of the target and the distractors.

Similar to experiment 1, we analyzed the mean angular deviations of the saccades by carrying out a series of RANOVAs. The first analysis focused on the effect of reward (in the cases where reward was unambiguous), with factors for modalities (visual, auditory, bimodal same side, bimodal opposite side) and reward (high vs low) and the second analysis focused on contrasting reward congruency and spatial congruency in the bimodal distractors, with factors for visual reward (high vs low), auditory reward (high vs low), and spatial congruency of the visual and auditory distractors (same vs opposite). In all RANOVAs, an extra factor time was included to model preconditioning to postconditioning changes in the angular deviation of the saccades. All statistical procedures (e.g., computation of effect sizes, *post hoc* tests) were identical to those used in experiment 1.

## Results of experiment 2

### Conditioning part

The mean correct responses were 99.4% across 160 trials, with an SD of 1%.

### Saccadic localization of distractors (calibration task)

**Figure 3.**
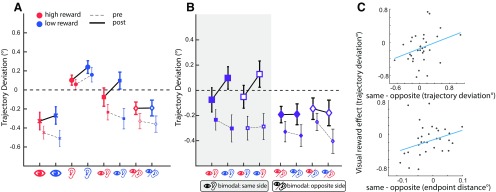
The effect of reward value on saccade trajectories in experiment 2. ***A***, Baseline corrected saccadic trajectory deviations in high- versus low-reward value conditions of visual, auditory, bimodal/same-side and bimodal/opposite-sides. The thin error bars and the dashed connecting line between them correspond to the preconditioning part, and the thick error bars and the solid connecting line between them indicate the data of the postconditioning part. A RANOVA revealed a significant main effect of reward in Post compared with Pre across all modalities (see the main text for details). ***B***, Baseline corrected saccadic trajectory deviation in all bimodal conditions. ***C***, Analysis of bimodal conditions shown in *B* showed a significant effect of visual reward. Two follow-up robust regression analyses revealed that the size of this effect (shown on the Y-axis) was reliably predicted by the magnitude of perceived difference between bimodal stimuli on the same versus opposite sides. The contrast between the two bimodal conditions (bimodal same side − bimodal opposite side) was calculated as the difference in trajectory deviations of these conditions in Pre (shown on the X-axis of top panel) and the difference in the end point distance of saccades measured during an independent saccadic localization task performed before the associative learning (shown on the X-axis of bottom panel). Error bars indicate the SEM.

In a saccadic localization task, we asked participants to report the perceived location of the distractors by making a saccadic eye movement to them. The main purpose of this task was to estimate how participants localized the bimodal stimuli. Estimations of how well participants discriminated between bimodal stimuli on the same and opposite sides, derived from an independent saccadic localization task, could then be used as a predictor of the observed effects in the main task (see the Results of experiment 2; Fig. 3*C*).

The data files of one participant in this task were not properly stored by the eye tracker, and therefore this participant was removed from the following analyses (*N* = 31). Comparison of the distance of the end point of the saccade from the center of the visual distractor (i.e., *X* = 3.6° and *Y* = 0) revealed that participants localized bimodal stimuli on the same and opposite sides to the same location as the visual distractors (the median distance in visual, bimodal same side and bimodal opposite side conditions were: 0.36° ± 0.1°, 0.34° ± 0.1°, 0.36° ± 0.11° before learning reward associations, and 0.41° ± 1.29°, 0.67 ° ± 1.77°, 0.68° ± 1.76° after learning reward associations). A RANOVA analysis with time (two levels: before and after learning reward associations), modality (three levels: visual, bimodal same side, bimodal opposite side) revealed no significant main or interaction effect (all *p* values > 0.1).

Since in our experiments auditory stimuli were not spatially localized and since localization of auditory stimuli is in general imprecise, we analyzed auditory stimuli separately. As expected, auditory stimuli were localized less accurately, as demonstrated by a larger distance of the end points of saccades from the center of the visual distractor (mean ± SD of the median distance across all trials in Pre: 5.99° ± 5.53°; in Post: 4.05° ± 5.35°). There was a significant reduction in the end point distance of the saccade from Pre to the Post (*p* = 0.002, Cohen’s *d* = 0.6). This finding is likely due to the fact that after extensive training and experience with co-occurring audiovisual stimuli, auditory distractors were assumed to be localized to the same location as the visual distractors.

### Saccadic task (main task)

Based on the exclusion criteria (identical to those in experiment 1), 5% of the trials were excluded in the Pre, and 4% of the trials were excluded in the Post.

#### Reward associations across different modalities

To examine whether reward value has a significant effect on saccade deviations after learning of reward associations, we next performed a RANOVA analysis with time (two levels: Pre and Post), modality (four levels: visual, auditory, bimodal same side, bimodal opposite side), and reward (two levels: high and low) as independent factors and angular deviations of saccades as the dependent factor (Fig. 3*A*). Note that in this analysis only conditions with unambiguous reward values (i.e., reward-congruent conditions) were included. RANOVA (lower bound corrected) revealed a significant main effects of time (Pre vs Post: *F*_(1,31)_ = 11.353, *p* = 0.002, $\eta_{p}^{2}$ = 0.26) and modality (*F*_(3,93)_ = 26.73, *p* = 1.31 × 10^−5^, $\eta_{p}^{2}$ = 0.46). The main effect of time corresponds to significantly larger trajectory deviations in Pre (mean ± SD across all modalities, −0.24 ± 0.3) compared with Post (mean ± SD, −0.07 ± 0.3), indicating that training on the task leads to a reduction in active inhibition of distractor locations and hence decreased trajectory deviations of saccades.

The significant main effect of modality corresponded to a difference across modalities in the magnitude of trajectory deviations of saccades with visual distractors causing the most deviated saccades away from the distractors (mean ± SD: Pre = −0.47 ± 0.35; Post = −0.29 ± 0.44), followed by bimodal distractors on the opposite side (Pre = −0.4 ± 0.4; Post = −0.19 ± 0.34) and bimodal distractors on the same side (Pre = −0.26 ± 0.48; Post = 0.01 ± 0.48). Auditory distractors caused deviations toward the distractors (Pre = 0.11 ± 0.31; Post = 0.17 ± 0.29). *Post hoc* tests for the main effect of modality revealed significant differences between auditory and all other distractor conditions (all *p* values <0.001, all Cohen’s *d* values >0.8) and between visual and bimodal distractors on the same side (*p* = 0.0009, Cohen’s *d* = 0.75).

Most importantly, the RANOVA also revealed a significant interaction between reward and time (*F*_(3,93)_ = 5.5402, *p* = 0.02, $\eta_{p}^{2}$ = 0.15). *Post hoc* analysis revealed that whereas after associative learning high-reward distractors led to significantly stronger trajectory deviations compared with low-reward stimuli (mean ± SD: −0.12 ± 0.32 and −0.03 ± 0.34 for high and low reward, respectively; *p* = 0.047, Cohen’s *d* = 0.36), no difference between these conditions was observed in the preconditioning phase (mean ± SD: −0.24 ± 0.31 and −0.25 ± 0.31 for high and low reward, respectively, *p* = 0.79, Cohen’s *d* = 0.04). This result demonstrates that reward associative learning changes the perceived salience of distractors across all modalities, and that this change in salience cannot be due to the differences in the physical attributes of stimuli before the associative learning.

#### Reward effects in bimodal conditions: the effect of spatial congruency and reward congruency

We next focused on the reward effects in bimodal stimuli (Fig. 3*B*). A four-way RANOVA with time (Pre vs Post), visual reward (high vs low), auditory reward (high vs low), and spatial location (same vs opposite sides) as factors was performed on the data of all bimodal conditions. This analysis (lower-bound correction) revealed a significant main effect of time (*F*_(1,31)_ = 24.292, *p* = 2.63 × 10^−5^ $\eta_{p}^{2}$ = 0.44) corresponding to a significant reduction in the interference of distractors in Post compared with Pre (mean ± SD across all bimodal conditions: −0.30 ± 0.36 and 0.07 ± 0.31 in Pre and Post, respectively). Moreover, significant interactions were found between spatial location and time (*F*_(1,31)_ = 5.68, *p*= 0.023, $\eta_{p}^{2}$ = 0.15), and between visual reward and time (*F*_(1,31)_ = 4.81, *p* = 0.035, $\eta_{p}^{2}$ = 0.13). As mentioned in the previous section, the significant effect of time suggests that training on the task leads to a reduction in the interference of distractors. This change in the curvature of saccades across time was stronger for bimodal stimuli with auditory and visual components on the same side compared with opposite sides (mean ± SD of angular deviations in Pre compared with Post: −0.28 ± 0.42 vs 0.02 ± 0.45 for same side and −0.33 ± 0.38 vs −0.17 ± 0.34 for opposite sides, respectively). Accordingly, *post hoc* tests for the significant interaction between spatial location and time showed that whereas before learning of reward values saccade curvatures of bimodal same and opposite sides were not different (*p* = 0.35, Cohen’s *d* = 0.16), after associative learning these conditions significantly diverged from each other (*p* = 0.024, Cohen’s *d* = 0.42), perhaps because participants paid more attention to the spatial characteristics of reward-associated sounds and visual stimuli.

Likewise, the significant interaction between visual reward and time was further examined by *post hoc* tests comparing bimodal distractors comprising a high-reward compared with a low-reward visual stimulus in Post and Pre. There was a trend for a significant difference between bimodal distractors with visual high-reward stimuli compared with low-reward stimuli in Post (mean ± SD: −0.12 ± 0.30 and −0.03 ± 0.37, respectively; *p* = 0.08, Cohen’s *d* = 0.31), whereas in Pre the differences were in the opposite direction and nonsignificant (mean ± SD: −0.28 ± 0.34 and −0.33 ± 0.46 for high and low visual reward, respectively, *p* = 0.35, Cohen’s *d* = 0.16). Although the difference between visual high- and low-reward stimuli in Post did not reach statistical significance, the significant interaction between visual reward and time indicates that the influence of visual reward on saccade trajectories significantly increased after associative learning. Furthermore, the effect of visual reward did not depend on whether bimodal stimuli were on the same or opposite sides (*F*_(1,31)_ = 1.57, *p* = 0.22, $\eta_{p}^{2}$ = 0.048, for the three-way interactions among visual reward, side, and time), indicating that across all bimodal conditions, the perceived salience of the bimodal distractors is predominantly determined by the reward value of the visual stimuli. One explanation for the dominance of visual reward is that the reward information of auditory stimuli is disregarded once a substantial conflict in spatial positions of the two modalities is detected. To examine this possibility, we next tested whether the strength of the influence of visual reward was correlated with the degree to which participants discriminated between bimodal stimuli on the same and opposite-sides before the conditioning. Note that, as discussed above, saccade trajectory deviations did not significantly differ between these bimodal conditions in Pre. Nevertheless, for each individual the amount of difference between bimodal-same and bimodal-opposite sides could reveal how well that participant could discriminate between the two configurations. To this end, two robust regression analyses were performed. The first analysis (Fig. 3*C*, top) examined the degree to which the difference in saccade deviations of same-side versus opposite-side bimodal stimuli in Pre (i.e., a proxy of how well participants discriminated the spatial characteristics of the two types of stimuli) predicted the strength of the visual reward effect (i.e., the difference in trajectory deviations of all bimodal conditions comprising a high-reward visual stimulus and those comprising a low-reward visual stimulus in Post while correcting for the differences in Pre). This analysis revealed a positive slope (regression slope = 0.35, *t*_(30)_ = 2.13, *p* = 0.041), indicating that the effect of visual reward was stronger in participants who were more sensitive to the difference in spatial alignment of bimodal stimuli. To ensure that this effect is not driven by intrinsic correlations between saccadic parameters of different distractor conditions in the main task, we performed a second regression analysis, where the data from an independent task (saccade localization task performed before learning reward associations) was used to derive a measure of the discrimination between bimodal stimuli (Fig. 3*C*, bottom). To this end, we calculated the median distance of the end points of the saccades (relative to the visual distractor location) in bimodal same and opposite sides. Again, we found a significant positive slope between the magnitude of the difference between bimodal same and opposite sides and the visual reward effect (regression slope = 2.32, *t*_(29)_ = 2.06, *p* = 0.046). These results indicate that the more a conflict in the spatial information between the two modalities was perceived, the higher the weight that was assigned to the visual compared with the auditory reward value became.

## Discussion

In the present study, our aim was to examine the integration of reward value across sensory modalities during a visually guided saccade task. More specifically, we examined whether pavlovian conditioning has an effect similar to those in the previous studies that used transient rewards, and further examined whether factors such as reward congruency and spatial congruency would influence the integration of learned reward values. In experiment 1, we showed that the reward values learned via pavlovian conditioning permeated the stimuli even during the nonreward part, with a significant effect of reward value on the trajectory deviations of visual distractors and a significant effect of the value congruence in bimodal distractors. In experiment 2, we reconfirmed the effect of pavlovian conditioning on the following nonreward part as there was a significant effect of reward value across all modalities, which exclusively occurred after learning of reward associations compared with a preconditioning phase. Importantly, after extensive training with bimodal stimuli and experiencing stimuli that were clearly misaligned and those that were not, bimodal distractors were predominantly modulated by the visual reward value.

### Pavlovian conditioning leads to long-lasting changes in perceived salience of reward associated stimuli

Our experimental paradigm was similar to a previous study that specifically examined the impact of transient rewards of visual distractors (i.e., the color of a target associated with high or low reward value in a previous trial could serve as a distractor on next, switch trials) on saccadic trajectories (Hickey and van Zoest, 2012). However, in our study, stimulus features were never shared between the target and the distractors, and moreover, the distractors acquired their associative values through a separate pavlovian reward-conditioning task. It is therefore, remarkable that completely task-irrelevant distractors that were never rewarded during the oculomotor task could still compete with the processing of the target and lead to changes in the saccade trajectories.

This pattern of result is in line with a series of recent studies showing that associative reward learning can enhance the salience of rewarded stimuli and lead to value-driven attentional capture, even in the following nonreward phase (Anderson et al., 2011; Theeuwes and Belopolsky, 2012; Yantis et al., 2012; Chelazzi et al., 2013; Hickey and van Zoest, 2013; Bucker and Theeuwes, 2018; Mine and Saiki, 2018). The impact of associative value during the nonreward phase depends on whether the previously rewarded stimuli serve as targets or distractors. Accordingly, stronger attentional capture by high-reward distractors can interfere with the task goals and decrease performance. Our results extend these previous findings to the domain of cross-modal associative reward value.

### Reward value guides multisensory integration during oculomotor planning

In audiovisual integration, there are several bottom–up and top–down factors that help the brain decide whether audio and visual information should be integrated or segregated (Chen and Spence, 2017; but see Cao et al., 2019 for neural correlates). Over decades, numerous studies have shown that stimuli spatial misalignment and temporal asynchrony (Stein and Meredith, 1993) are the main bottom–up factors that disrupt cross-modal integration. Nevertheless, recent studies show emerging evidence that top–down factors such as expectations of stimulus characteristics (Van Wanrooij et al., 2010), emotional and motivational factors (Bruns et al., 2014), and semantic congruence (Doehrmann and Naumer, 2008) can all influence audiovisual integration (Macaluso et al., 2016). When incongruent information is provided by auditory and visual signals in the same event, conflicts may be resolved based on their individual modality precision or modality appropriateness (Welch and Warren, 1980) or the system would call for cognitive control (Pessoa, 2009).

Our results are in line with previous studies suggesting a role of top–down factors in cross-modal binding. In experiment 1, participants were asked to perform the saccadic task without prior experience with our experimental bimodal stimuli. Furthermore, the associated reward values of the visual and auditory stimuli were learned separately, without them being bound as a unity in the conditioning part. Consequently, during postconditioning phase top–down information related to the congruence in associated reward values was instrumental in determining whether co-occurring visual and auditory signals are likely to have a common source. This resulted in the significant effect of reward congruency in experiment 1, which is similar to the findings of a recent study (Sanz et al., 2018), demonstrating the importance of cross-modal semantic congruency in determining the perceptual salience of audiovisual stimuli.

Unlike experiment 1, in experiment 2, participants were asked to perform a localization task and the saccadic task before the conditioning part; hence, they were already exposed to the bimodal stimuli even before learning the reward associations of the stimuli in each modality respectively, and had extensive practice for the saccadic task. Moreover, since auditory stimuli were presented either on the same or the opposite side to the visual distractors, their reliability in predicting a common source for the two modalities was reduced. Accordingly, in experiment 2 the information related to the congruence in reward value was neither novel nor informative for deciding whether unisensory signals had common or separate sources. This led to downweighting of the auditory reward and dominance of visual reward information. The correlation of the visual reward effect with the sensitivity of participants in detecting a spatial misalignment in bimodal stimuli supports the idea that once a strong conflict in spatial relationships of unisensory signals is detected, visual reward wins over the auditory reward. This flexible weighting of reward-related information across sensory modalities is akin to the findings of previous studies that investigated the balance between multisensory integration and unisensory dominance by manipulating physical characteristics of stimuli, demonstrating the pivotal role of the reliability of the signals (Yuval-Greenberg and Deouell, 2009; Rohe and Noppeney, 2015, 2018).

When a multisensory object is encountered, our brain should solve a binding problem, deciding whether inputs from different sensory modalities emanate from the same multisensory object or not (Körding et al., 2007). In doing so, different types of signals related to the physical properties of stimuli (e.g., their spatial and temporal overlap) or their high-level associative or task-related properties (reward or attention) can inform the brain of whether signals should be integrated or remain segregated. We show a gradient of integration based on the reward values that is strongest when auditory and visual signals are likely to originate from the same object and are congruent in their reward values, and weakest when a spatial misalignment is introduced leading to the dominance of visual rewards. We propose that the integration of reward values across sensory modalities that we observed follows the principles of Bayesian cue integration (Knill and Pouget, 2004; Angelaki et al., 2009). Statistically optimal, Bayesian integration has been repeatedly shown to underlie the integration of information across senses (Ernst and Banks, 2002; Alais and Burr, 2004; Ernst and Bülthoff, 2004). Bayes optimal integration entails a linear averaging between signals weighted by their reliability (i.e., inverse of the variance), resulting in the combined signal having a higher reliability than either of the individual inputs. Accordingly, if one of the input signals has higher uncertainty, more weight is assigned to the other signal. In line with this principle, we observed that whereas in experiment 1 reward values were combined across sensory modalities (demonstrated by an interaction effect between auditory and visual rewards), visual rewards dominated oculomotor responses once audition delivered unreliable information regarding the side of the audiovisual distractors (being either on the same side or opposite sides in experiment 2). This is further supported by showing that the strength of the dominance of visual rewards was correlated with the perceived misalignment of the auditory and visual stimuli (Fig. 3*C*). Assigning a higher weight to the reward values of vision, which in general has a higher precision in spatial localization (Welch and Warren, 1980), is an optimal strategy to deal with the uncertainty introduced by the spatial misalignment. We note, however, that our conclusion is preliminary since we did not systematically manipulate the spatial reliability of each individual unisensory signal in our current study. This could be done, for instance, by introducing different degrees of spatial noise to each signal (Heron et al., 2004). This way a gradual modulation of the weights assigned to the auditory and visual signals and their rewards as a function of their spatial uncertainty could be induced that allows the testing of predictions of a Bayes optimal integration model.

### Limitations and future directions

The strength of the trajectory deviations of saccades in the distractor paradigm used in our study depends on the spatial position of the target, the distractor, and most importantly the target–distractor spatial separation, being stronger for near (i.e., a small angular separation between target and distractor <20°) than far distractors (Van der Stigchel, 2010). Furthermore, previous studies have shown that the direction of saccade curvatures, being toward or away from the distractor, depends on the target–distractor spatial separation, with deviations toward mainly associated with close distractors and deviations away with far distractors (McSorley et al., 2009). In our experiments, visual distractors were presented on the horizontal meridian, thus having a 90° angular separation from the target location (always presented on the vertical meridian). Auditory distractors were lateralized to the left or right but were not spatially localized. Hence, both visual and auditory distractors were presented at a location that is not typically associated with the strongest saccadic deviation (i.e., far distractors). Nevertheless, we observed a significant deviation of the saccades for visual distractors, irrespective of the rewards. Auditory distractors had, however, no impact on their own, presumably because they were localized to even farther locations than visual distractors (as also supported by the saccadic localization task). An important direction for future studies is to test whether the reward modulation of the oculomotor responses to bimodal distractors depends on the strength of trajectory deviations produced by individual unimodal stimuli (e.g., compared between far and near distractors or between high- or low-saliency auditory and visual unisensory stimuli).

The literature on cross-modal interactions suggests that audiovisual binding is strongest when visual and auditory stimuli are completely overlapping, in time as well as in space (Stein and Meredith, 1993). In our experiments, however, auditory and visual stimuli were overlapping in time, but auditory stimuli were only lateralized to the left and right but were not spatially localized. While the lack of spatial localization may account for the relatively weak effect of auditory distractors on saccade trajectories, we note that previous studies that attempted to optimally localize auditory stimuli reported comparable effect sizes for trajectory deviations of far auditory and bimodal distractors (Doyle and Walker, 2002; Walker and McSorley, 2008; Heeman et al., 2016). Furthermore, there are a number of naturalistic situations where auditory and visual stimuli are not completely colocalized but nevertheless coherent multisensory experience is elicited, as exemplified by the spatial ventriloquism effect (Chen and Vroomen, 2013). Our findings show that in these latter cases reward value can be used as an additional source of information to decide whether unisensory signals should be combined or separated. Notwithstanding, the lack of spatial localization might have resulted in an underestimation of the reward-related effects during oculomotor planning, and future studies will be needed to determine whether and how spatial localization of sounds impacts on the reward effects observed in our study. Moreover, further research is required to know whether the effects we observed are generalizable to other tasks (e.g., perceptual or value-based decision tasks), training procedures (e.g., training with operant conditioning, assignment of reward to combined audiovisual stimuli as opposed to separate reward values assigned to each modality), and task-contingence of reward (e.g., task-relevant vs task-irrelevant rewards).

### Possible neural underpinnings

Distractors composed of different sensory modalities had been shown to have an influence on the curvature of the saccades made to a target (Doyle and Walker, 2002; Campbell et al., 2010; Heeman et al., 2016), and we provide evidence for the modulation of these effects by cross-modal reward value. Deviation of saccades toward or away from a visual distractor is proposed to be due to the top–down inhibition of the distractor-evoked responses at the level of superior colliculus, a midbrain structure that determines saccade vectors (Munoz et al., 2000). This inhibition could lead to the concurrent inhibition of neural populations that program the saccades to the target and are under a common motor map with the distractor (McPeek et al., 2003; McPeek, 2006). Our current results suggest that cross-modal reward may provide top–down information for such inhibition. Given the dense connectivity between the brain structures that encode reward value in basal ganglia and superior colliculus (Hikosaka et al., 2014), it is likely that cross-modal integration of reward value occurs at the level of superior colliculus. Future studies are needed to explore this possibility.

### Conclusion

In conclusion, our results from the two experiments demonstrate that in a saccadic task that highly relies on the processing of visual spatial information, the reward values from a different sensory modality that does not render reliable spatial information can still be integrated with the reward value of the visual modality. The weighting of reward information depends on the assumptions regarding the source of visual and auditory stimuli. In case of uncertainty regarding the spatial colocalization, the congruence of reward value guides the audiovisual interactions. However, if a conflict in spatial information of unisensory signals is perceived, the modality that is more task relevant, is assigned with a higher weight for its reward information.

## Synthesis

Reviewing Editor: Li Li, New York University Shanghai Decisions are customarily a result of the Reviewing Editor and the peer reviewers coming together and discussing their recommendations until a consensus is reached. When revisions are invited, a fact-based synthesis statement explaining their decision and outlining what is needed to prepare a revision will be listed below. The following reviewer(s) agreed to reveal their identity: Paola Binda. Note: If this manuscript was transferred from JNeurosci and a decision was made to accept the manuscript without peer review, a brief statement to this effect will instead be what is listed below.

Both reviewers think that the ms addresses an interesting and important question in the field. However, the ms cannot be considered for publication before the authors satisfactorily addresses the reviewers’ concerns listed below.

Reviewer 1

In the current study, Pooresmaeili et al., used an oculomotor task in which the subjects’ saccade eye traces were influenced by a visual distracter. The sign of the distorted traces, and the magnitude associated with the reward were consistent with previous findings. The novelty in the current work is that the authors now introduced another sensory modality cue-auditory distracter that was also associated with the reward value. Unfortunately, the effect from the auditory distracter on the oculormotor traces was negligible. However, the authors found that the value congruency between the visual and auditory cue has a statistically significant effect, although this effect is quite small. The authors further found that when the visual and auditory distracters were congruent in space, that is, from the same side, then reward information seemed to be integrated across the cues, particularly when this reward information was learned prior to the task. Overall, the topic of the multisensory interaction in a reward associated oculormotor task is interesting, representing a step further in the field. However, the statistic effects were marginal.

Concerns:

The auditory distracter does not generate any significant effect, thus it is not surprising the visual cue is always dominant. Has the auditory cue been fully explored, for example, in terms of its spatial locations, frequency and magnitude, to see whether itself can also affect the oculomotor trace as the visual distracter?

Reviewer 2

he paper addresses an important, relatively unexplored issue: whether and how we integrate reward value across modalities.

It does so with an interesting and rigorous method: exploiting an involuntary behaviour (bypassing issues related to response bias) measured by tracking gaze position (a quantitative and precise measure).

I have two main suggestions (minor points, related to the presentation of the results)

1-the authors introduce the issue of multisensory integration in general, as developed to predict the integration of sensory signals. however, it is very unclear (to me at least) which principles may rule multisensory integration of reward value. It would be very important to describe possible models e.g. statistically optimal or Bayesian integration, super-additivity, ... - either in the opening of the paper (laying out predictions to be tested by the experiments), or at the end of it (discussing the principles that best account for the results).

2-the results of experiment 2 are presented in the same format as those of experiment 1; however, since here there is an additional pre-conditioning test where different stimuli (e.g. visual and auditory) produce different effects, I wonder whether analysing the results of reward by looking at post-pre conditioning differences could help clarify the picture. For example, it looks like taking the post-pre difference would reinstate the significant effect of reward value on visual information (thereby replicating experiment 1).

I feel that clarifying these two issues would make the interpretation of the results easier and thereby more accessible to the very broad and diverse audience that will be interested in the reported results

minor:

first paragraph of the introduction & reference list: Libera and Chelazzi, 2006 - I think it is *della* Libera and Chelazzi 2006

## Author Response

Response to the Reviewers

Synthesis of Reviews:

Computational Neuroscience Model Code Accessibility Comments for Author (Required):

n.a.

Significance Statement Comments for Author (Required):

n.a.

Comments on the Visual Abstract for Author (Required):

n.a.

Synthesis Statement for Author (Required):

Both reviewers think that the ms addresses an interesting and important question in the field. However, the ms cannot be considered for publication before the authors satisfactorily addresses the reviewers' concerns listed below.

We thank the editor for giving us the opportunity to revise our manuscript and for the overall positive evaluation of our work. The editor's guidance for the revision and the reviewers' comments have been most valuable and helpful. We have addressed the issues highlighted by the editor and the reviewers in detail. Specifically, reviewer 1 raised concerns regarding the fact that in our paradigm auditory distractors did not produce significant trajectory deviations on their own, which may account for the dominance of visual reward effects in bimodal distractors. We have clarified that although auditory distractors did not lead to strong trajectory deviations, their reward values modulated the overall salience of audiovisual distractors in experiment 1. Only in experiment 2, where a misalignment was introduced between auditory and visual stimuli, reward value of vision dominated the oculomotor responses. The reviewer also suggested several factors that may account for the lack of strong trajectory deviations in auditory distractors. We could rule out some of these factors (such as the frequency of tones) but others such as the spatial location of the sounds have not been tested in the current study. We trust however, that our results provide testable prediction for future studies where the factors proposed by the reviewer can be systematically examined and compared against the results and effect sizes that are reported here. Reviewer 2 was very positive about our study and made suggestions to improve the clarity of the manuscript by explaining the possible mechanisms that could underlie the integration of reward values across sensory modalities. To answer the reviewers' suggestions and concerns we adapted the manuscript at multiple locations, by adding information related to the interpretation of the results and limitations of our conclusions. Below is a point-by-point reply to the issues raised by both reviewers. We believe that these changes have made our paper stronger and we hope that our manuscript will now be suitable for publication in the current form.

The editors' and reviewers' comments are in bold, our responses are in normal font. Changes to the manuscript based on reviewers' suggestions are marked in red, in the manuscript document. Text excerpts from the modified manuscript are marked in italics and referred to by their corresponding page numbers.

Reviewer comments:

Reviewer 1

In the current study, Pooresmaeili et al., used an oculomotor task in which the subjects' saccade eye traces were influenced by a visual distracter. The sign of the distorted traces, and the magnitude associated with the reward were consistent with previous findings. The novelty in the current work is that the authors now introduced another sensory modality cue-auditory distracter that was also associated with the reward value. Unfortunately, the effect from the auditory distracter on the oculormotor traces was negligible. However, the authors found that the value congruency between the visual and auditory cue has a statistically significant effect, although this effect is quite small. The authors further found that when the visual and auditory distracters were congruent in space, that is, from the same side, then reward information seemed to be integrated across the cues, particularly when this reward information was learned prior to the task. Overall, the topic of the multisensory interaction in a reward associated oculormotor task is interesting, representing a step further in the field. However, the statistic effects were marginal.

Concerns:

The auditory distracter does not generate any significant effect, thus it is not surprising the visual cue is always dominant. Has the auditory cue been fully explored, for example, in terms of its spatial locations, frequency and magnitude, to see whether itself can also affect the oculomotor trace as the visual distracter?

We thank the reviewer for the overall positive evaluation of our study, and are grateful for the reviewer's suggestions to clarify the impact of auditory distractors. We agree with the reviewer that in our study auditory stimuli on their own had negligible effects on the saccade curvatures. Nevertheless, we show that when presented concurrently with visual distractors, reward values of auditory distractors affect trajectory deviations caused by the audiovisual stimuli in Experiment 1. In this experiment, we found an interaction effect between visual and auditory reward values, as trajectory deviations were significantly larger when reward values from the two modalities were congruent (i.e. both High or both Low) compared to when they were incongruent (i.e. one modality High and the other Low value, Figure 2C). This interaction effect, albeit with a small effect size as the reviewer has pointed out, indicates that the reward value of auditory distractors and its congruence with the visual rewards influences the overall response to an audiovisual object. Since in this experiment participants had no prior experience with the audiovisual stimuli (as there was no Pre-conditioning phase), congruency in reward values provided an additional source of information regarding whether the concurrent auditory and visual inputs emanated from the same object. In Experiment 2 however, participants had prior experience with audiovisual distractors and most importantly, they experienced, in some instances, a conflict in the spatial position of auditory and visual inputs (i.e. auditory and visual stimuli were presented either on the same or on the opposite sides). This spatial conflict reduced the reliability of auditory distractors in predicting a common source for concurrent auditory and visual inputs and accordingly we observed a dominance of visual reward information (please also see the reply to the point 1 of reviewer 2 and the information added to the revised manuscript on pages 22-23, lines 631-660). As shown in Figure 3C, the dominance of visual reward was correlated with the degree to which participants perceived this spatial incongruency (i.e. a difference between audiovisual distractors on the same- and opposite-sides) prior to the main task. Taken together, these results show that in Experiment 1 reward values were combined across sensory modalities, as lack of prior experience with audiovisual stimuli and lack of a strong incongruence in the spatial position of auditory and visual stimuli strengthened the belief that they belonged to the same object, although to a lesser degree when their reward values were incongruent. In contrast, introducing a spatial conflict in experiment 2, to which participants were exposed prior to the main task, reduced the reliability and informativeness of auditory reward values and led to the dominance of visual rewards.

We fully agree with the reviewer that the impact of auditory distractors should be further explored before these results could be generalized to other contexts. In line with the suggestions of the reviewer, we predict that the spatial location is the most critical factor determining the strength of oculomotor responses to the auditory distractors. In general, the strength of saccades' curvature in the distractor paradigm employed in our study depends on the spatial position of the target, the distractor and most importantly the target-distractor spatial separation, being stronger for near (i.e. a small angular separation between target and distractor <20{degree sign}) than far distractors (Van der Stigchel, 2010). Furthermore, previous studies have shown that the direction of saccade curvatures, being towards or away from the distractor, also depends on the target-distractor spatial separation, with deviations towards mainly associated with close distractors and deviations away with far distractors (McSorley et al., 2009). In our experiments, visual distractors were presented on the horizontal meridian, thus having a 90{degree sign} angular separation from the target location (always presented on the vertical meridian). Auditory distractors were lateralized to the left or right but were not spatially localized. Hence, both visual and auditory distractors were not presented at a location that produces a strong competition between the target and the distractor and thus a large saccadic deviation. Nevertheless, we observed a significant deviation of the saccades for visual distractors, irrespective of the rewards. Auditory distractors had however no impact on their own, presumably because they were localized to even farther locations than visual distractors (as also supported by the saccadic localization task reported on page 15, line 447-444, please also see below). Two important modifications to our paradigm could be undertaken by future studies to reveal the extent to which the effects we observed is generalized across tasks. Firstly, the spatial position of the unimodal distractors can be systematically manipulated to test whether the reward modulations of bimodal stimuli depend on the strength of trajectory deviations of unimodal stimuli (e.g. compared between far and near distractors or between faint and salient stimuli). Secondly, auditory stimuli that are spatially co-localized to the same position as visual distractors should be compared with non-localized sounds that we used here. We have thus far not undertaken these manipulations and hence cannot draw conclusions whether our effects are generalizable to or perhaps even stronger in these settings. We predict however, that in line with the reviewer's intuition using auditory distractors that produce stronger saccade curvatures on their own may lead to stronger effect sizes for the reward effects in bimodal stimuli.

In our pilot studies, we used a range of sound frequencies and did not observe a systematic difference between them in terms of the strength of saccade deviations of auditory distractors. We have not manipulated the magnitude/intensity of the sounds in this study and cannot rule out the possibility that they may have an impact. We note however that our study is the first to explore the interaction of reward values from different sensory modalities in determining the salience of bimodal stimuli, and we show that this interaction could also occur between unimodal stimuli that on their own are weakly competing with a saccade target. The reviewer's proposal for a possibility of the dependence of the effect size of reward effects in bimodal distractors on the salience of the individual unimodal stimuli is a very interesting direction for future research. We have now added the information related to these point in Methods section on page 6, lines 172-179:

“The auditory pure tones in both of our experiments had an intensity of 55 dB and frequencies of 600 or 1000 Hz. We decided for these frequencies since they produce approximately the same loudness based on their location on equal-loudness contours (Robinson and Dadson, 1956). In our pilot experiments we tested a range of sound frequencies but did not observe a difference in terms of the strength of saccade trajectory deviations induced by auditory distractors in the main saccade task. The intensity of the sounds was selected based our subjective estimation of a sound level that is most comfortable for the participants but we did not explore the effect of different sound intensities in terms of their effect on saccade curvatures.”

And in the Discussion on Page 23 under Limitations and future directions heading (lines 662-678):

“The strength of the saccadic trajectory deviations in the distractor paradigm employed in our study depends on the spatial position of the target, the distractor and most importantly the target-distractor spatial separation, being stronger for near (i.e. a small angular separation between target and distractor <20{degree sign}) than far distractors (Van der Stigchel, 2010). Furthermore, previous studies have shown that the direction of saccade curvatures, being towards or away from the distractor, depends on the target-distractor spatial separation, with deviations towards mainly associated with close distractors and deviations away with far distractors (McSorley et al., 2009). In our experiments, visual distractors were presented on the horizontal meridian, thus having a 90{degree sign} angular separation from the target location (always presented on the vertical meridian). Auditory distractors were lateralized to the left or right but were not spatially localized. Hence, both visual and auditory distractors were presented at a location that is not typically associated with the strongest saccadic deviation (i.e. far distractors). Nevertheless, we observed a significant deviation of the saccades for visual distractors, irrespective of the rewards. Auditory distractors had however no impact on their own, presumably because they were localized to even farther locations than visual distractors (as also supported by the saccadic localization task). An important direction for future studies is to test whether the reward modulation of the oculomotor responses to bimodal distractors depends on the strength of trajectory deviations produced by individual uni-modal stimuli (e.g. compared between far and near distractors or between high- or low-saliency auditory and visual uni-sensory stimuli).”

Reviewer 2

the paper addresses an important, relatively unexplored issue: whether and how we integrate reward value across modalities.

It does so with an interesting and rigorous method: exploiting an involuntary behaviour (bypassing issues related to response bias) measured by tracking gaze position (a quantitative and precise measure).

We thank the reviewer very much for the very positive evaluation of our paper and we hope that the current revision will make the paper even better.

I have two main suggestions (minor points, related to the presentation of the results)

1-the authors introduce the issue of multisensory integration in general, as developed to predict the integration of sensory signals. however, it is very unclear (to me at least) which principles may rule multisensory integration of reward value. It would be very important to describe possible models e.g. statistically optimal or Bayesian integration, super-additivity, ... - either in the opening of the paper (laying out predictions to be tested by the experiments), or at the end of it (discussing the principles that best account for the results).

We are very grateful to the reviewer for bringing this up. Although we had discussed our findings in relation to the previous research showing that audiovisual integration follows the principles of Bayesian cue integration, where signals are weighted based on their reliability (Knill and Pouget, 2004; Rohe and Noppeney, 2015, 2018), we had not explicitly laid out the mechanisms that underlie our effects. We have now made this explicit in the Discussion section of the revised manuscript. We decided to add this point to the Discussion since our original hypothesis was in fact a dominance of vision in all cases during a visually-driven saccadic task, since such a task critically depends on the processing of spatial information where vision has the highest precision (see the last paragraph of the Introduction). However, we observed an interaction effect between the reward values of visual and auditory stimuli in experiment 1, indicating that the reward values of both modalities are taken into account when planning an eye movement in the presence of an audiovisual distractor. When auditory stimuli delivered conflicting information regarding the spatial position of distractors in Experiment 2 however, a higher weight was assigned to the visual rewards and auditory reward information was completely disregarded. The flexible weighting of reward information based on the reliability of their spatial information that we observed across the two experiments suggests that a statistically optimal, Bayesian inference model may underlie our reported effects. We note however, that this conclusion is preliminary and future studies with more systematic manipulations of spatial and reward uncertainties of the two sensory modalities will be needed to confirm our conclusion. This information is now added to the page 22 and 23 (lines 631-660):

“When a multisensory object is encountered, our brain should solve a binding problem, deciding whether inputs from different sensory modalities emanate from the same multisensory object or not (Körding et al., 2007). In doing so, different types of signals related to the physical properties of stimuli (e.g. their spatial and temporal overlap) or their high-level associative or task-related properties (reward or attention) can inform the brain of whether signals should be integrated or remain segregated. We show a gradient of integration based on the reward values that is strongest when auditory and visual signals are likely to originate from the same object and are congruent in their reward values, and weakest when a spatial misalignment is introduced leading to the dominance of visual rewards. We propose that the integration of reward values across sensory modalities that we observe follows the principles of Bayesian cue integration (Knill and Pouget, 2004; Angelaki et al., 2009). Statistically optimal, Bayesian integration has been repeatedly shown to underlie the integration of information across senses (Ernst and Banks, 2002; Alais and Burr, 2004; Ernst and Bülthoff, 2004). Bayes optimal integration entails a linear averaging between signals weighted by their reliability (i.e. inverse of the variance), resulting in the combined signal having a higher reliability than either of the individual inputs. Accordingly, if one of the input signals has higher uncertainty, more weight is assigned to the other signal. In line with this principle, we observed that whereas in Experiment 1 reward values were combined across sensory modalities (demonstrated by an interaction effect between auditory and visual rewards), visual rewards dominated oculomotor responses once audition delivered unreliable information regarding the side of the audiovisual distractors (being either on the same- or opposite-sides in Experiment 2). This is further supported by showing that the strength of the dominance of visual rewards was correlated with the perceived misalignment of the auditory and visual stimuli (Figure 3C). Assigning a higher weight to the reward values of vision, which in general has a higher precision in spatial localization (Welch and Warren, 1980), is an optimal strategy to deal with the uncertainty introduced by the spatial misalignment. We note however, that our conclusion is preliminary since we did not systematically manipulate the spatial reliability of each individual uni-sensory signal in our current study. This could be done, for instance, by introducing different degrees of spatial noise to each signal (Heron et al., 2004). This way a gradual modulation of the weights assigned to the auditory and visual signals and their rewards as a function of their spatial uncertainty could be induced that allows to test predictions of a Bayes optimal integration model”.

2-the results of experiment 2 are presented in the same format as those of experiment 1; however, since here there is an additional pre-conditioning test where different stimuli (e.g. visual and auditory) produce different effects, I wonder whether analysing the results of reward by looking at post-pre conditioning differences could help clarify the picture. For example, it looks like taking the post-pre difference would reinstate the significant effect of reward value on visual information (thereby replicating experiment 1).

We thank the reviewer for this suggestion. Two methods can be used to control for the differences between stimuli prior to the conditioning (i.e. during Pre). One method as suggested by the reviewer is to subtract the saccade trajectories of pre- from post-, thus removing differences between conditions that are due to factors other than rewards (e.g. difference in color, luminance, sound pitch or loudness). The second method is to account for difference in pre by using it as a factor in our statistical models. We decided to apply the second method for the statistical analysis of the data but illustrate saccade trajectories without any subtraction so that the direction (away and towards) of trajectory deviations in both phases (pre and post) can be readily examined (as in Figure 3). To this end, the statistical analysis of the data from experiment 2 was performed by accounting for differences in pre- as noted by the reviewer: all RANOVAs included an extra factor Time in order to model the pre-post differences (see page 15, lines 408-416). This way we controlled for differences between reward conditions prior to the conditioning. Hence, the effect of reward across all modalities reported on Page 17 (lines 470-478) is corrected for differences in pre (i.e. we show a significant interaction between the factors Reward and Time showing and our posthoc tests confirm that the main effect of reward was only present in post- and not in pre-). Since we did not observe an interaction effect between reward and modality, further pairwise comparisons between reward conditions of individual modalities (visual, auditory and bimodal conditions) was not statistically warranted. At the request of the reviewer, we performed paired ttests between reward conditions of each modality subtracting pre-conditioning deviations from each condition. This analysis revealed a significant difference between high and low reward conditions only in bimodal stimuli on the same side (p = 0.004) while none of the other comparisons reached statistical significance (all ps>0.1). Thus in experiment 2, we reproduce the effect of reward on saccade trajectories, albeit this effect is significant across all modalities but does not reach statistical significance for every individual modality. Possible reasons for this pattern of results are the prolonged training with the saccadic task that diminishes the trajectory deviations in general and also more variation in distractor condition (i.e. having visual, auditory, bimodal same-side and bimodal-opposite sides in experiment 2 compared to visual, auditory and bimodal same-side in experiment 1). The latter could have led to a reduction in the salience of reward information in visual, unimodal distractors, which form a lower proportion of all distractor conditions in experiment 2 compared to experiment 1. Nevertheless, the significant effect of reward across modalities, which is in the same direction as the reported effects of reward in visual, unimodal distractors in previous studies and in experiment 1 of the current study, supports a significant reward-related change in distractors' salience from pre- to post- in experiment 2.

I feel that clarifying these two issues would make the interpretation of the results easier and thereby more accessible to the very broad and diverse audience that will be interested in the reported results

We thank the reviewer for these valuable suggestions and we hope that we could satisfactorily clarify these issues.

minor:

first paragraph of the introduction & reference list: Libera and Chelazzi, 2006 - I think it is *della* Libera and Chelazzi 2006

We thank the reviewer for pointing out this unintended mistake. We have now corrected this citation.

References

Alais D, Burr D (2004) The Ventriloquist Effect Results from Near-Optimal Bimodal Integration. Curr Biol 14:257-262.

Angelaki DE, Gu Y, DeAngelis GC (2009) Multisensory integration: psychophysics, neurophysiology, and computation. Curr Opin Neurobiol 19:452-458.

Ernst MO, Banks MS (2002) Humans integrate visual and haptic information in a statistically optimal fashion. Nature 415:429.

Ernst MO, Bülthoff HH (2004) Merging the senses into a robust percept. Trends Cogn Sci 8:162-169.

Heron J, Whitaker D, McGraw PV (2004) Sensory uncertainty governs the extent of audio-visual interaction. Vision Res 44:2875-2884.

Knill DC, Pouget A (2004) The Bayesian brain: the role of uncertainty in neural coding and computation. Trends Neurosci 27:712-719.

Körding KP, Beierholm U, Ma WJ, Quartz S, Tenenbaum JB, Shams L (2007) Causal inference in multisensory perception. PLoS One 2:e943.

McSorley E, Cruickshank AG, Inman LA (2009) The development of the spatial extent of oculomotor inhibition. Brain Res 1298:92-98.

Robinson DW, Dadson RS (1956) A re-determination of the equal-loudness relations for pure tones. Br J Appl Phys 7:166-181.

Rohe T, Noppeney U (2015) Sensory reliability shapes perceptual inference via two mechanisms. J Vis 15:22.

Rohe T, Noppeney U (2018) Reliability-Weighted Integration of Audiovisual Signals Can Be Modulated by Top-down Attention. eNeuro 5.

Van der Stigchel S (2010) Recent advances in the study of saccade trajectory deviations. Vision Res 50:1619-1627.

Welch RB, Warren DH (1980) Immediate perceptual response to intersensory discrepancy. Psychol Bull 88:638.

## References

[B1] Alais D, Burr D (2004) The ventriloquist effect results from near-optimal bimodal integration. Curr Biol 14:257–262. 10.1016/j.cub.2004.01.029 14761661

[B2] Anderson BA, Laurent PA, Yantis S (2011) Value-driven attentional capture. Proc Natl Acad Sci USA U S A 108:10367–10371. 10.1073/pnas.1104047108 21646524 PMC3121816

[B3] Angelaki DE, Gu Y, DeAngelis GC (2009) Multisensory integration: Psychophysics, neurophysiology, and computation. Curr Opin Neurobiol 19:452–458. 10.1016/j.conb.2009.06.008 19616425 PMC2749464

[B4] Asutay E, Västfjäll D (2016) Auditory attentional selection is biased by reward cues. Sci Rep 6:36989. 10.1038/srep36989 27841363 PMC5107919

[B5] Bruns P, Maiworm M, Röder B (2014) Reward expectation influences audiovisual spatial integration. Atten Percept Psychophys 76:1815–1827. 10.3758/s13414-014-0699-y 24874263

[B6] Bucker B, Theeuwes J (2018) Stimulus-driven and goal-driven effects on Pavlovian associative reward learning. Vis Cogn 26:131–148. 10.1080/13506285.2017.1399948

[B7] Calvert GA (2001) Crossmodal processing in the human brain: Insights from functional neuroimaging studies. Cereb Cortex 11:1110–1123. 10.1093/cercor/11.12.1110 11709482

[B8] Campbell KL, Al-Aidroos N, Fatt R, Pratt J, Hasher L (2010) The effects of multisensory targets on saccadic trajectory deviations: Eliminating age differences. Exp Brain Res 201:385–392. 10.1007/s00221-009-2045-5 19851761 PMC3399904

[B9] Cao Y, Summerfield C, Park H, Giordano BL, Kayser C (2019) Causal inference in the multisensory brain. Neuron 102:1076–1087.e8. 10.1016/j.neuron.2019.03.043 31047778

[B10] Chandrasekaran C (2017) Computational principles and models of multisensory integration. Curr Opin Neurobiol 43:25–34. 10.1016/j.conb.2016.11.002 27918886 PMC5447489

[B11] Chelazzi L, Perlato A, Santandrea E, Della Libera C (2013) Rewards teach visual selective attention. Vision Res 85:58–72. 10.1016/j.visres.2012.12.005 23262054

[B12] Chelazzi L, Eštočinová J, Calletti R, Lo Gerfo E, Sani I, Della Libera C, Santandrea E (2014) Altering spatial priority maps via reward-based learning. J Neurosci 34:8594–8604. 10.1523/JNEUROSCI.0277-14.2014 24948813 PMC6608215

[B13] Chen L, Vroomen J (2013) Intersensory binding across space and time: A tutorial review. Atten Percept Psychophys 75:790–811. 10.3758/s13414-013-0475-4 23709064

[B14] Chen Y-C, Spence C (2017) Assessing the role of the “unity assumption” on multisensory integration: A review. Front Psychol 8:445. 10.3389/fpsyg.2017.00445 28408890 PMC5374162

[B15] Colavita FB (1974) Human sensory dominance. Percept Psychophys 16:409–412. 10.3758/BF03203962

[B16] Corneil BD, Van Wanrooij M, Munoz DP, Van Opstal AJ (2002) Auditory-visual interactions subserving goal-directed saccades in a complex scene. J Neurophysiol 88:438–454. 10.1152/jn.2002.88.1.438 12091566

[B17] Della Libera C, Chelazzi L (2006) Visual selective attention and the effects of monetary rewards. Psychol Sci 17:222–227. 10.1111/j.1467-9280.2006.01689.x 16507062

[B18] Della Libera C, Chelazzi L (2009) Learning to attend and to ignore is a matter of gains and losses. Psychol Sci 20:778–784. 10.1111/j.1467-9280.2009.02360.x 19422618

[B19] Doehrmann O, Naumer MJ (2008) Semantics and the multisensory brain: How meaning modulates processes of audio-visual integration. Brain Res 1242:136–150. 10.1016/j.brainres.2008.03.071 18479672

[B20] Doyle MC, Walker R (2002) Multisensory interactions in saccade target selection: Curved saccade trajectories. Exp Brain Res 142:116–130. 10.1007/s00221-001-0919-2 11797089

[B21] Engelmann J, Damaraju E, Padmala S, Pessoa L (2009) Combined effects of attention and motivation on visual task performance: Transient and sustained motivational effects. Front Hum Neurosci 3:4. 10.3389/neuro.09.004.2009 19434242 PMC2679199

[B22] Ernst MO, Banks MS (2002) Humans integrate visual and haptic information in a statistically optimal fashion. Nature 415:429–433. 10.1038/415429a 11807554

[B23] Ernst MO, Bülthoff HH (2004) Merging the senses into a robust percept. Trends Cogn Sci 8:162–169. 10.1016/j.tics.2004.02.002 15050512

[B24] Gilbert CD, Sigman M (2007) Brain states: Top-down influences in sensory processing. Neuron 54:677–696. 10.1016/j.neuron.2007.05.019 17553419

[B25] Heeman J, Nijboer TCW, Van der Stoep N, Theeuwes J, Van der Stigchel S (2016) Oculomotor interference of bimodal distractors. Vision Res 123:46–55. 10.1016/j.visres.2016.04.002 27164053 PMC4894297

[B26] Heron J, Whitaker D, McGraw PV (2004) Sensory uncertainty governs the extent of audio-visual interaction. Vision Res 44:2875–2884. 10.1016/j.visres.2004.07.001 15380993

[B27] Hickey C, van Zoest W (2012) Reward creates oculomotor salience. Curr Biol 22:R219–R220. 10.1016/j.cub.2012.02.007 22497933

[B28] Hickey C, van Zoest W (2013) Reward-associated stimuli capture the eyes in spite of strategic attentional set. Vision Res 92:67–74. 10.1016/j.visres.2013.09.008 24084197

[B29] Hikosaka O, Kim HF, Yasuda M, Yamamoto S (2014) Basal ganglia circuits for reward value-guided behavior. Annu Rev Neurosci 37:289–306. 10.1146/annurev-neuro-071013-013924 25032497 PMC4148825

[B30] Kersten D, Mamassian P, Yuille A (2004) Object perception as Bayesian inference. Annu Rev Psychol 55:271–304. 10.1146/annurev.psych.55.090902.142005 14744217

[B31] Knill DC, Pouget A (2004) The Bayesian brain: The role of uncertainty in neural coding and computation. Trends Neurosci 27:712–719. 10.1016/j.tins.2004.10.007 15541511

[B32] Körding KP, Beierholm U, Ma WJ, Quartz S, Tenenbaum JB, Shams L (2007) Causal inference in multisensory perception. PLoS One 2:e943. 10.1371/journal.pone.0000943 17895984 PMC1978520

[B33] Lakens D (2013) Calculating and reporting effect sizes to facilitate cumulative science: A practical primer for t-tests and ANOVAs. Front Psychol 4:863. 10.3389/fpsyg.2013.00863 24324449 PMC3840331

[B34] Leo F, Noppeney U (2014) Conditioned sounds enhance visual processing. PLoS One 9:e106860. 10.1371/journal.pone.0106860 25192387 PMC4156410

[B35] Luque D, Beesley T, Morris R, Jack BN, Griffiths O, Whitford T, Le Pelley ME (2017) Goal-directed and habit-like modulations of stimulus processing during reinforcement learning. J Neurosci 37:3009–3017. 28193692 10.1523/JNEUROSCI.3205-16.2017 PMC6596732

[B36] Macaluso E, Driver J (2005) Multisensory spatial interactions: A window onto functional integration in the human brain. Trends Neurosci 28:264–271. 10.1016/j.tins.2005.03.008 15866201

[B37] Macaluso E, Noppeney U, Talsma D, Vercillo T, Hartcher-O’Brien J, Adam R (2016) The curious incident of attention in multisensory integration: Bottom-up vs. top-down. Multisens Res 29:557–583. 10.1163/22134808-00002528

[B38] Maiworm M, Bellantoni M, Spence C, Röder B (2012) When emotional valence modulates audiovisual integration. Atten Percept Psychophys 74:1302–1311. 10.3758/s13414-012-0310-3 22623217

[B39] Manohar SG, Chong TT-J, Apps MAJ, Batla A, Stamelou M, Jarman PR, Bhatia KP, Husain M (2015) Reward pays the cost of noise reduction in motor and cognitive control. Curr Biol 25:1707–1716. 10.1016/j.cub.2015.05.038 26096975 PMC4557747

[B40] McPeek RM (2006) Incomplete suppression of distractor-related activity in the frontal eye field results in curved saccades. J Neurophysiol 96:2699–2711. 10.1152/jn.00564.2006 16885521 PMC1876735

[B41] McPeek RM, Han JH, Keller EL (2003) Competition between saccade goals in the superior colliculus produces saccade curvature. J Neurophysiol 89:2577–2590. 10.1152/jn.00657.2002 12611995

[B42] McSorley E, Cruickshank AG, Inman LA (2009) The development of the spatial extent of oculomotor inhibition. Brain Res 1298:92–98. 10.1016/j.brainres.2009.08.081 19733156

[B43] Mine C, Saiki J (2018) Pavlovian reward learning elicits attentional capture by reward-associated stimuli. Atten Percept Psychophys 80:1083–1095. 10.3758/s13414-018-1502-2 29542094

[B44] Mohanty A, Gitelman DR, Small DM, Mesulam MM (2008) The spatial attention network interacts with limbic and monoaminergic systems to modulate motivation-induced attention shifts. Cereb Cortex 18:2604–2613. 10.1093/cercor/bhn021 18308706 PMC2567423

[B45] Munoz DP, Dorris MC, Paré M, Everling S (2000) On your mark, get set: Brainstem circuitry underlying saccadic initiation. Can J Physiol Pharmacol 78:934–944. 11100942

[B46] Nyström M, Holmqvist K (2010) An adaptive algorithm for fixation, saccade, and glissade detection in eyetracking data. Behav Res Methods 42:188–204. 10.3758/BRM.42.1.188 20160299

[B47] Pessoa L (2009) How do emotion and motivation direct executive control? Trends Cogn Sci 13:160–166. 10.1016/j.tics.2009.01.006 19285913 PMC2773442

[B48] Pooresmaeili A, FitzGerald THB, Bach DR, Toelch U, Ostendorf F, Dolan RJ (2014) Cross-modal effects of value on perceptual acuity and stimulus encoding. Proc Natl Acad Sci U S A 111:15244–15249. 10.1073/pnas.1408873111 25288729 PMC4210286

[B49] Robinson DW, Dadson RS (1956) A re-determination of the equal-loudness relations for pure tones. Br J Appl Phys 7:166–181. 10.1088/0508-3443/7/5/302

[B50] Rohe T, Noppeney U (2015) Sensory reliability shapes perceptual inference via two mechanisms. J Vis 15(5):22 1–16. 10.1167/15.5.22 26067540

[B51] Rohe T, Noppeney U (2018) Reliability-weighted integration of audiovisual signals can be modulated by top-down attention. eNeuro 5:ENEURO.0315-17.2018 10.1523/ENEURO.0315-17.2018 PMC5844059 29527567

[B52] Sanz LRD, Vuilleumier P, Bourgeois A (2018) Cross-modal integration during value-driven attentional capture. Neuropsychologia 120:105–112. 10.1016/j.neuropsychologia.2018.10.014 30342964

[B53] Small DM, Gitelman D, Simmons K, Bloise SM, Parrish T, Mesulam M-M (2005) Monetary incentives enhance processing in brain regions mediating top-down control of attention. Cereb Cortex 15:1855–1865. 10.1093/cercor/bhi063 15746002

[B54] Spence C (2009) Explaining the Colavita visual dominance effect. Prog Brain Res 176:245–258. 19733761 10.1016/S0079-6123(09)17615-X

[B55] Stein BE, Meredith MA (1993) The merging of the senses. Cambridge, MA: MIT.

[B56] Stein BE, Stanford TR (2008) Multisensory integration: Current issues from the perspective of the single neuron. Nat Rev Neurosci 9:255–266. 10.1038/nrn2331 18354398

[B57] Theeuwes J, Belopolsky AV (2012) Reward grabs the eye: Oculomotor capture by rewarding stimuli. Vision Res 74:80–85. 10.1016/j.visres.2012.07.024 22902641

[B58] Van der Stigchel S (2010) Recent advances in the study of saccade trajectory deviations. Vision Res 50:1619–1627. 10.1016/j.visres.2010.05.028 20553739

[B59] Van Wanrooij MM, Bremen P, John Van Opstal A (2010) Acquired prior knowledge modulates audiovisual integration. Eur J Neurosci 31:1763–1771. 10.1111/j.1460-9568.2010.07198.x 20584180

[B60] Walker R, McSorley E (2008) The influence of distractors on saccade-target selection: Saccade trajectory effects. J Eye Mov Res 2:1–13.

[B61] Welch RB, Warren DH (1980) Immediate perceptual response to intersensory discrepancy. Psychol Bull 88:638–667. 7003641

[B62] Yantis S, Anderson BA, Wampler EK, Laurent PA (2012) Reward and attentional control in visual search. Nebr Symp Motiv 59:91–116. 10.1007/978-1-4614-4794-8_5 23437631 PMC4323078

[B63] Yuval-Greenberg S, Deouell LY (2009) The dog’s meow: Asymmetrical interaction in cross-modal object recognition. Exp Brain Res 193:603–614. 10.1007/s00221-008-1664-6 19066869

[B64] Zuanazzi A, Noppeney U (2018) Additive and interactive effects of spatial attention and expectation on perceptual decisions. Sci Rep 8:6732. 10.1038/s41598-018-24703-6 29712941 PMC5928039

